# Vitamin A Deficiency in the Early-Life Periods Alters a Diversity of the Colonic Mucosal Microbiota in Rats

**DOI:** 10.3389/fnut.2020.580780

**Published:** 2020-12-04

**Authors:** Baolin Chen, Shu Liu, Di Feng, Lu Xiao, Ting Yang, Tingyu Li, Wuqing Sun, Jie Chen

**Affiliations:** ^1^Chongqing Key Laboratory of Child Nutrition and Health, Children's Nutrition Research Center, Children's Hospital of Chongqing Medical University, Chongqing, China; ^2^Ministry of Education Key Laboratory of Child Development and Disorders, Chongqing, China; ^3^China International Science and Technology Cooperation Base of Child Development and Critical Disorders, Chongqing, China; ^4^Information Technological Service Center, Children's Hospital of Chongqing Medical University, Chongqing, China

**Keywords:** gut mucosal microbiota, pregnancy, lactation, weaning period, vitamin A deficiency (VAD)

## Abstract

Vitamin A deficiency (VAD) remains a public health issue worldwide, affecting pregnant women and children. The early-life microbiota is a potentially effective intervention target for modulating immune and metabolic development of the host. This paper investigates the effects of VAD during different life periods on the structure of the colonic mucosa microbiota in adolescent rats. The results showed that the concentrations of serum retinol were > ~1.05 μmol/L in maternal VA normal (VAN)rats and < 0.7 μmol/L in maternal VAD rats, while the serum retinol levels were higher than 0.7 μmol/L in the pups of the VAN group and below 0.5 μmol/L in the pups of the VAD group. Compared to the offspring persistent with VAN from embryonic stage (group A), all the remaining groups exhibited an increased ratio of *Firmicutes*/*Bacteroidetes* abundance. A metagenome analysis (LEfSe) and a differentially abundant features approach using Metastats for genus abundances revealed that *Diaphorobacter* and *Psychrobacter* were increased in the offspring persistent with VAD from embryonic stage (group B);*Bifidobacterium* was decreased and *Staphylococcus* was increased in the offspring with VAD after weaning (group C); *Propionibacterium* and *Enterobacter* were increased significantly in the offspring with VAD during gestation(group E); and *Ochrobactrum* was increased in group B and the offspring with VAD during gestation and lactation(group D). *Faecalibacterium* abundance was significantly and positively related to serum retinol levels, while that of *Staphylococcus* was significantly and negatively correlated with serum retinol levels. VAD in different life periods can alter the gut microbiome in rats, but VAD in the early-life periods (especially gestation and/or lactation) leads to a diversity of the colonic mucosal microbiota in adolescent rats as well as an imbalance of the ratio between *Firmicutes* and *Bacteroidetes*. The early-life period may become a time window of VA intervention to improve intestinal microbiota caused by VA deficiency, but the specific mechanism requires more in-depth research.

## Introduction

Vitamin A (VA) is an organic nutrient that is essential for human embryonic development and homeostasis (1). For vitamin A deficiency (VAD), one of the three major micronutrient deficiencies announced by the WHO, the main affected populations are children and pregnant women in low-income and middle-income countries. It was estimated that 29% of children younger than 5 years suffer from VAD and that for 2% of the children who died in this age group in sub-Saharan Africa and Southern Asia, fatality was due to VAD (2).

Van de Pavert et al. (3) found that in the mice, VA obtained from the mother's diet can regulate the size of lymph nodes in unborn offspring. Offspring fed a VAD diet showed obvious lymph node shrinkage and decreased immune response efficiency. Our previous studies have successfully constructed VAD and VA normal model in Sprague Dawley (SD) rats (4), and found that VAD rats during gestation can reduce the percentages of CD11c^+^ dendritic cells and CD4^+^CD25^+^ T lymphocytes in the intestinal Peyer's patch of offspring and affect the homing of B cells in mesenteric lymph nodes and that VAD during early-life stages of rats (gestation and lactation) can significantly reduce the proportion of CD8^+^ intestinal epithelial lymphocytes in pups (5). Therefore, VAD, especially during the early stages of life, has a significant impact on the growth and development of offspring.

The intestinal microbiota is an important internal environmental factor for human health. The normal gut microbiota imparts specific functions to host nutrient metabolism, maintenance of structural integrity of the gut mucosal barrier, immunomodulation, cognitive and behavioral development and protection against pathogens (6).Throughout the 1st year of life, the gut microbiota increases dramatically in diversity and stability and reportedly reaches an adult-like configuration in the subsequent years (7). Emerging evidence suggests that the colonization of microbes in the human body during the early-life period plays a critical role in the establishment and maturation of developmental pathways and that disruption of this optimal microbial succession may contribute to lifelong and intergenerational deficits in growth and development (8). So far, more and more rat models were used to study changes of intestinal microbiota (9).

Some studies have shown that VAD can cause changes in the intestinal microbiota that affect the normal function of the body. Lee and Ko (10) had found that VA exerted an antiviral effect on Norovirus infection by altering the composition of the intestinal microbiota. Hibberd et al. while using gnotobiotic mice, found that VAD had the largest effect among tested factors on microbial community and meta-transcriptome, with increases in *Bacteroides vulgatus* in the context of VA deprivation, which are results that could have important implications for bile acid metabolisms (11). However, little is known regarding at which period the impact of VAD has the greatest effect on the gut microbiota in adolescent rats. Samples used for studying the intestinal microbiota are mainly feces, but many studies had shown that the feces is not enough to represent the entire gastrointestinal-localized microbiota, and that even the luminal microbiota and the mucosa-associated microbes of the same intestine are different (12). Therefore, to explore which stage of life VAD has the greatest impact on the colonic mucosal microbiota in this study, we constructed a rat model of VAD at different stages of development and analyzed the effects of VAD on the colonic mucosal microbiota structure to better evaluate the colonization status of the gut microbiota.

## Materials and Methods

### Animals, Diets, and Sample Collection

This study was approved by the Animal Experimentation Ethical Committee of Chongqing Medical University (Chongqing, China) and complied with the principles of good laboratory animal care. The 4-wk-old specific pathogen-free (SPF) SD rats were obtained from the Experimental Animal Center of Chongqing Medical University. As shown in Supplementary Figure 1, first, 4-wk-old female SD rats were randomly selected and divided equally into two groups, namely, maternal VAN and maternal VAD. The maternal VAD rats were fed a VAD diet that contained 400 IU of VA/kg for 4 wk to construct a VAD animal model before gestation, whereas the maternal VAN rats received VAN food that contained 6,500 IU of VA/kg as a control (13). The male rats were fed with VAN food. When the serum retinol levels of blood samples taken from the tails of the maternal VAD rats decreased to 0.7 μmol/L and those of the maternal VAN rats increased to 1.05 μmol/L, the female rats were mated with normal male rats. Pregnant maternal rats were fed either the VAD diet or the VAN diet during both gestation and lactation to maintain stable serum retinol levels. These rats were housed in the same room with a constant airflow system, controlled temperature (22–24°C), and a 12 h light/dark cycle. The SPF animal house was certified for experimental animals [SYXK (Yu) 2017-0012]).

There were 5 VAN and 5 VAD pregnant rats in each group, and the average litter size was 8–10 per litter. After a weaning period of 21 postnatal days, pups were fed continuously for 3 wk with the VAN diet and designated as group A or with the VAD diet and designated as group B. Pups of the gestational VAN rats were fed the VAD diet for 3 wk after they were weaned (group C), whereas pups of the gestational VAD rats were fed the VAN diet for 3 wk after weaning (group D). After birth, VAD pups were cross-fostered to VAN dams during the lactation period and then fed the VAN diet after weaning (group E), and VAN pups were cross-fostered to VAD dams during the lactation period and then fed the VAD diet after weaning (group F). Next, the offspring rats were sacrificed at 6 weeks old, and blood was collected immediately from the femoral artery to test serum retinal concentration. The colons of rats in each group were extracted and stored at −80°C for further study after cleaning with 0.01 mol/L PBS. All surgery was performed under sodium pentobarbital (30 mg/kg) anesthesia, and euthanasia was accomplished with CO_2_.

### Serum Retinol Detection

Serum retinol concentrations were measured using high-performance liquid chromatography (HPLC) according to our previously described methods (14), with slight modifications. Briefly, 200 mL of serum was deproteinized with dehydrated alcohol, and then retinol was extracted with hexane and evaporated with nitrogen gas. The residue of retinol was dissolved in 100 μL of the mobile phase mixture (methanol: water = 97:3). Finally, the prepared sample was measured using an HPLC apparatus (DGU-20As, Shimadzu Corporation, Japan) equipped with a C18 analytical column and a 315 nm ultraviolet photodiode array detector.

### DNA Extraction and 16S rDNA Amplicon Pyrosequencing

Total microbial genomic DNA samples were extracted from colon samples using a DNeasyPowerSoil Kit (QIAGEN, Inc., Netherlands) following the manufacturer's instructions and stored at −20°C prior to further analysis. The quantity and quality of extracted DNAs were measured using a NanoDrop ND-1000 spectrophotometer (Thermo Fisher Scientific, Waltham, MA, USA) and agarose gel electrophoresis, respectively.

PCR amplification of the bacterial 16S rRNA gene V4–V5 regions was performed using the forward primer 520F (5'- AYTGGGYDTAAAGNG-3') and the reverse primer 802R (5′-TACNVGGGTATCTAATCC-3′). Sample-specific 7-bp barcodes were incorporated into the primers for multiplex sequencing. The PCR components consisted of 5 μL of Q5 reaction buffer (5 ×), 5 μL of Q5 High-Fidelity GC buffer (5 ×), 0.25 μL of Q5 High-Fidelity DNA Polymerase (5 U/μl), 2 μL (2.5 mM) of dNTPs, 1 μL (10 μM) of each forward and reverse primer, 2 μL of DNA template, and 8.75 μL of ddH_2_O. Thermal cycling consisted of initial denaturation at 98°C for 2 min followed by 25 cycles consisting of denaturation at 98°C for 15 s, annealing at 55°C for 30 s, and extension at 72°C for 30 s, with a final extension of 5 min at 72°C. PCR amplicons were purified with AgencourtAMPure Beads (Beckman Coulter, Indianapolis, IN) and quantified using a PicoGreen dsDNA Assay Kit (Invitrogen, Carlsbad, CA, USA). After an individual quantification step, amplicons were pooled in equal amounts, and paired-end 2 × 300 bp sequencing was performed using the Illumina MiSeq platform with a MiSeq Reagent Kit v3 at Shanghai Personal Biotechnology Co., Ltd.

### Processing of Sequencing Data

Briefly, raw fastq files were quality-filtered using QIIME (v1.8.0) with the following criteria: (1) the reads were truncated at any site receiving an average quality score <20 over a 10 bp sliding window, discarding the truncated reads that were shorter than 150 bp; (2) primers were exactly matched allowing 1 nucleotide mismatching, and reads containing ambiguous characters were removed; and (3) only sequences that had overlaps longer than 10 bp were merged according to their overlap sequence. Reads that could not be assembled were discarded. Paired-end reads were assembled using FLASH (15).

The high-quality sequences were clustered into operational taxonomic units (OTUs) at 97% sequence identity by UCLUST (16). A representative sequence was selected from each OTU using default parameters. OTU taxonomic classification was conducted by BLAST searching the representative sequences set against the Greengenes database (17) using the best hit (18). To standardize the comparisons of the microbiota to avoid bias in the following analysis, samples were rarefied to 15,673 reads per sample. OTU-level alpha diversity indices such as the Chao1 richness estimator, abundance-based coverage estimator (ACE) metric, Shannon diversity index, and Simpson index were calculated using the OTU table in QIIME. A paired *t*-test or Wilcoxon's signed rank test was used for comparisons of the Chao1, ACE, Shannon, and Simpson indices between the control and experimental groups. A rarefaction curve was analyzed to measure whether the sequencing depth was reasonable. OTU-level ranked abundance curves were generated to compare the richness and evenness of OTUs among samples. An unweighted UniFrac distance metric analysis using the OTUs, a principal component analysis (PCA), was applied to reflect the differences and distances among the samples in the different groups. To more effectively distinguish between the two groups, PLS-DA (partial least squares discriminant analysis) was conducted using the mixOmics package. Taxon abundances at the phylum and genus levels were statistically compared among groups by Metastats (19). LEfSe (linear discriminant analysis effect size) was performed to detect differentially abundant taxa across groups using the default parameters (20). LDA scores were used to measure the contribution of each taxon to the significant differences. A genus whose LDA score was > 2 was designated as having a significant impact on the group. The sequence information was deposited in NCBI's Sequence Read Archive with the accession number PRJNA 615186.

### Statistical Analyses

Repeated measures analysis of variance, Student's *t*-test, Spearman's rank correlation and Kruskal-Wallis test analyses were performed using SPSS ver. 21.0 for Windows. Linear correlation analyses were applied between variables. Significance was accepted at *P* < 0.05.

## Results

### Serum Retinol Levels of Maternal Rats and Their Pups at Different Periods

**Figure 1:**
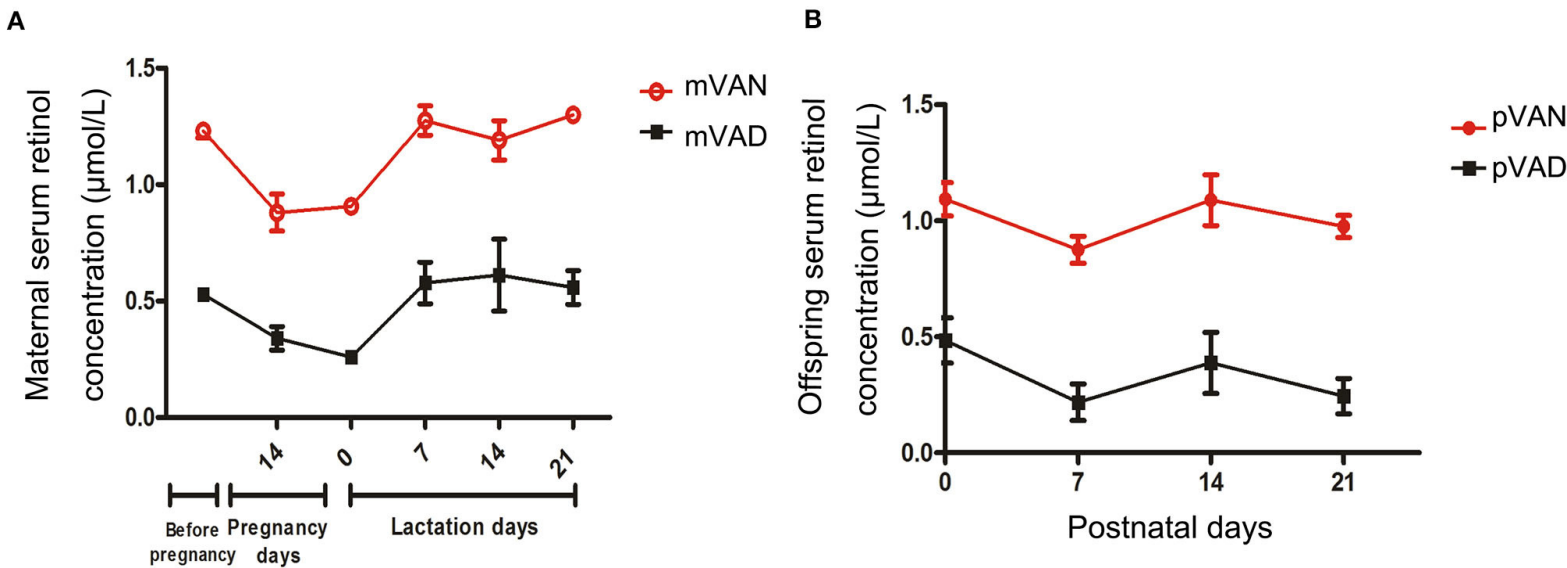
The levels of serum retinol in the mother rats and their offsprings with the different VA nutritional levels during each periods. **(A)** The comparison of serum retinol levels in the mother rats with VAN and VAD; **(B)** The comparison of serum retinol levels in the pups with VAN and VAD. mVAN refers to the maternal rats with VAN diet; mVAD refers to the maternal rats with VAD diet; pVAN refers to the pups of the mVAN; pVAD refers to the pups of the mVAD.

To evaluate VA nutritional levels in the early-life period, we monitored dynamic changes of serum retinol levels in maternal rats during gestational and lactational periods and pups from postnatal day 1 to weaning. The serum retinol of maternal rats are displayed in Figure 1A. Although the retinol of the mother rats in the VAN group (>1.05 μmol/L)were significantly higher than those in the VAD group(<0.7 μmol/L) (*p* < *0.0001*, Repeated measures analysis of variance), we surprisingly found that all the serum retinol levels in the two groups gradually declined with prolongation of the gestation period and then increased rapidly after delivery. The serum retinol levels of the pups are shown in Figure 1B. The levels of serum retinol remained higher than 0.7 μmol/L in the pups of the VAN group (pVAN), while those in the pups in the VAD group (pVAD) were below 0.5 μmol/L (*p* < *0.0001*, Repeated measures analysis of variance), and there was a gradual downward trend of the serum retinol levels in both the VAN and VAD groups at postnatal day 7. The above data suggest that both the VAN and VAD models in maternal rats and their offspring successfully produce corresponding VA bioavailability in the periods from pre-gestation to post-weaning.

### Effect of Persistent VAD Beginning at Gestation on the Colonic Mucosal Microbiota in Rats

**Figure 2:**
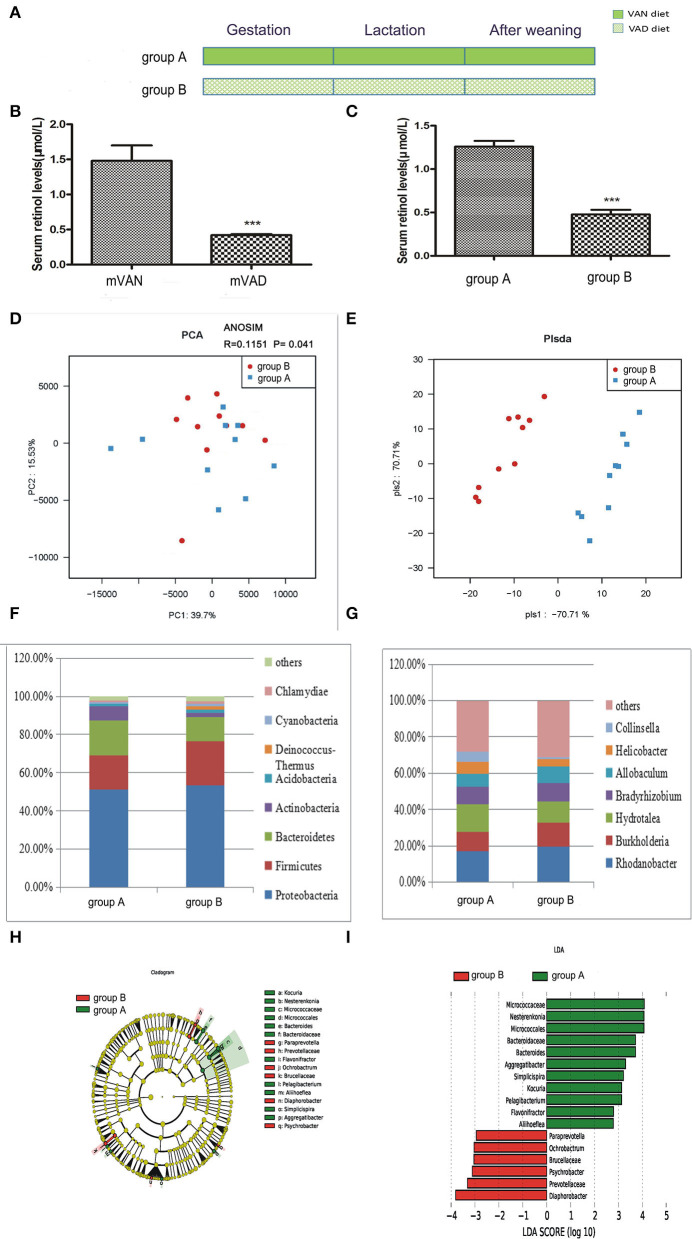
The comparison of colonic mucosal microbiota between the group A and group B. **(A)** Schematic diagram showing the two offspring groups: group A, group B. The group A pups were delivered and nursed by maternal VAN rats and then fed a VAN diet for 3 wk after weaning. The group B pups were birthed and nursed by maternal VAD rats and fed a VAD diet for 3 wk after weaning. **(B)** The serum retinal levels of the maternal rats. Serum VA levels were detected by HPLC. Values represent mean ± SD *n* = 10 mVAN and 10 mVAD, ***P < 0.001 by Student's *t*-test. mVAN refers to the maternal rats with VAN diet; mVAD refers to the maternal rats with VAD diet. **(C)** The serum retinal levels of offspring at 6 weeks old. Serum VA levels were detected by HPLC. Values represent mean ± SD *n* = 10 group A and 10 group B, ***P < 0.001 by Student's *t*-test. **(D,E)** The PCA and PLS-DA analysis of the samples between the group A and group B. **(D)** PCA scores were plotted based on the relative abundance of OTUs (*n* = 10). **(E)** PLS-DA was plotted based on the unweighted UniFrac distance metrics (*n* = 10). The red circles represent for the group B, blue squares for the group A. **(F)** The phylum level of dominant bacteria with relative abundance more than 1% in the two groups. **(G)** The genus level of dominant bacteria with relative abundance more than 5% in the two groups. **(H,I)** Different structures of colonic mucosal microbiota in offsprings rats with VAN or VAD diet by LEfSe analysis. **(H)** A cladogram of the statistical and biological differences in the colonic microbiota between the group A and group B, which are shown by the color of the most abundant phylotypes (red indicating group B, green A, and yellow non-significant) (*n* = 10). **(I)** A histogram of the LDA scores for the most abundant phylotypes (*n* = 10).

As shown in Figure 2, the rats were divided into two groups. The group A pups were delivered and nursed by maternal VAN rats and then fed a VAN diet for 3 wk after weaning, while the group B pups were delivered and nursed by maternal VAD rats and fed a VAD diet for 3 wk after weaning (Figure 2A). The serum retinol level in the mVAD mothers before copulation was significantly lower (0.42 ± 0.01 μmol/L) than that in the mVAN mothers (1.48 ± 0.22 μmol/L), which was a significant difference; *P* < *0.001* (Figure 2B). Furthermore, compared with that in the offspring in group A, the serum retinol level in the offspring in group B was significantly decreased (0.48 ± 0.05 μmol/L) (1.26 ± 0.07 μmol/L, *P* < *0.001*) (Figure 2C). The serum levels of retinol in the parental and offspring rats indicated that the animal model was successfully constructed. The colonic mucosa of the 6-wk-old offspring rats was used for subsequent analysis of microbial diversity.

After optimization, a total of 1,689,220 high-quality sequences were obtained from the 20 samples by MiSeq sequencing. We acquired numerous OTUs from valid sequences showing 97% similarity for further statistical analyses. Along with an increase in the amount of sequencing reads, the rarefaction curves of all the samples tended to be smooth. The approach of these rarefaction curves to the saturation plateau demonstrates that our sequencing data volume was reasonable for the present study (data not shown). The rank abundance curve visually depicts both species richness and species evenness. There were no significant differences in the length and steep gradient of the fold line between the A and B groups, indicating that there were no significant differences in community richness and community diversity metrics between the two groups (data not shown). In general, Chao1 and ACE diversity indices reflect the richness of the microbiota, while Shannon and Simpson diversity indices are considered indicators of community richness and evenness. After using the rank sum test, there were no significant differences in the Chao1, ACE and Shannon and Simpson diversity indices between the two groups.

To further assess differences in the colonic mucosal microbiota of rats with different VA levels, the unweighted UniFrac distance matrix was calculated according to the OTUs of each sample. PCA revealed a separation of the different VA levels based on the first two principal component (PC) scores, accounting for 39.7 and 15.53% of explained variances (Figure 2D). Meanwhile, ANOSIM analysis showed that the difference between the two groups was significantly greater than the difference within the group (*R* = 0.1151, *P* = 0.041, Figure 2D), indicating that our grouping was meaningful. In addition to PCA, PLS-DA clearly distinguished the samples of group A and group B based on the direction of the PLS-DA 1 axis, explaining −70.71% of the total variance (Figure 2E). These data suggest that there may be some differences in the distribution of the colonic mucosal microbiota.

Figure 2F shows the composition of the dominant microbiota between groups A and B at the phylum level. In group B, predominant phyla with a relative abundance of more than 1% were concentrated in the phyla *Proteobacteria* (53.38%), *Firmicutes* (23.15%), *Bacteroidetes* (12.71%), *Actinobacteria* (1.97%), *Acidobacteria* (1.93%), *Deinococcus-Thermus* (1.62%), *Cyanobacteria* (1.45%), and *Chlamydiae* (1.33%). In group A, predominant phyla with a relative abundance of more than 1% were concentrated in the phyla *Proteobacteria* (51.08%), *Bacteroidetes* (18.11%), *Firmicutes* (18.01%), *Actinobacteria* (7.52%), and *Acidobacteria* (1.4%). Compared with group A, group B exhibited increased abundances of the phyla *Proteobacteria, Firmicutes, Acidobacteria, Chlamydiae, Cyanobacteria*, and *Deinococcus-Thermus*, but the differences were not statistically significant (*P* > 0.05, Metastats), while the abundances of *Bacteroidetes* and *Actinobacteria* were slightly decreased (*P* > 0.05, Metastats).The *Firmicutes*/*Bacteroidetes* value of group A and group B was 1.21 ± 0.77 and 2.42 ± 0.86, respectively (*P* < *0.05*, Student's *t*-test), indicating that group B had intestinal microbiota disturbance at the phylum level. Figure 2G displays the composition of the dominant microbiota at the genus level. At the genus level, 5 genera accounted for more than 5% of the gut microbiota in group B, namely, *Rhodanobacter* (19.45%), *Burkholderia* (13.33%), *Hydrotalea* (11.88%), *Bradyrhizobium* (9.90%), and *Allobaculum* (9.27%). However, 7 genera accounted for more than 5% of the gut microbiota in group A, namely, *Rhodanobacter* (16.92%), *Hydrotalea* (15.12%), *Burkholderia* (10.79%), *Bradyrhizobium* (9.73%), *Allobaculum* (6.95%), *Helicobacter* (6.59%), and *Collinsella* (5.55%). Compared with those of group A, the abundances of the gut microbiota at the genus level that increased and decreased in group B were not significantly different (*P* > 0.05, Metastats).

A metagenome analysis approach (LEfSe) was used to identify the key phylotypes responsible for the differences between the two VA levels (Figures 2H,I). The absolute value of LDA ≥ 2 was defined as a significant influence on grouping. By comparing the colonic mucosal microbiota between groups A and B, there were 8 different genera in group A, namely, *Bacteroides* (LDA = 3.72, *P* = 0.03 < 0.05), *Pelagibacterium* (LDA = 3.13, *P* = 0.02), *Simplicispira* (LDA = 3.21, *P* = 0.03), *Flavonifractor* (LDA = 2.80, *P* = 0.03), *Aggregatibacter* (LDA = 3.30, *P* = 0.04), *Aliihoeflea* (LDA = 2.79, *P* = 0.047), *Kocuria* (LDA = 3.13, *P* = 0.04), and *Nesterenkonia* (LDA = 4.07, *P* = 0.03), and 4 different genera in group B, namely, *Ochrobactrum* (LDA = 3.04, *P* = 0.03), *Diaphorobacter* (LDA = 3.81, *P* = 0.02), *Psychrobacter* (LDA = 3.11, *P* = 0.04), and *Paraprevotella* (LDA = 2.95, *P* = 0.02).

### Effect of VAD After Weaning on the Colonic Mucosal Microbiota in Rats

**Figure 3:**
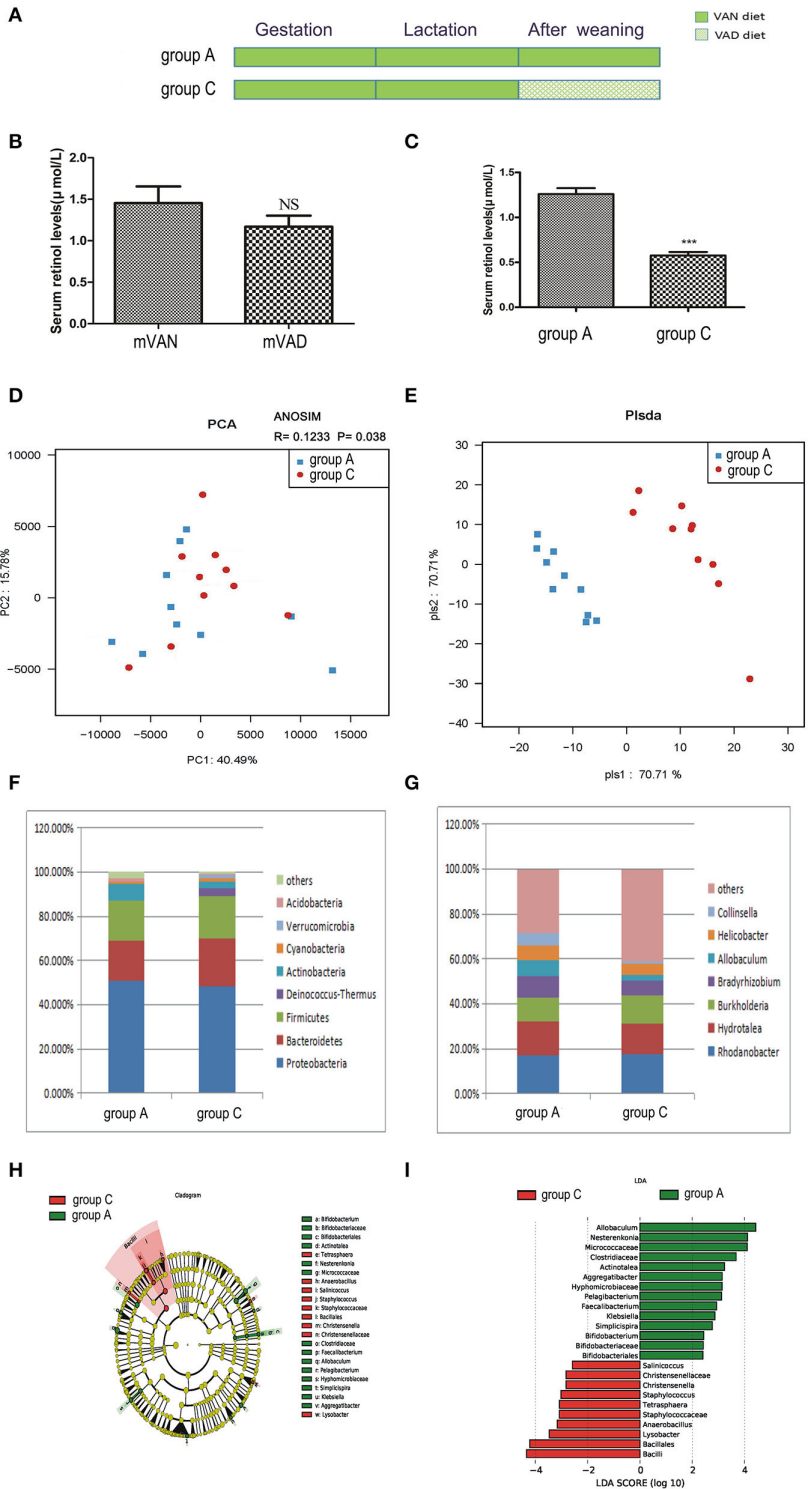
The comparison of colonic mucosal microbiota between the group A and group C. **(A)** Schematic diagram showing the two offspring groups: group A, group C. The group A pups were delivered and nursed by maternal VAN rats and then fed a VAN diet for 3 wk after weaning. The group C pups were birthed and nursed by maternal VAN rats and fed a VAD diet for 3 wk after weaning. **(B)** The serum retinal levels of maternal rats. Serum VA levels were detected by HPLC. Values represent mean ± SD. *n* = 10 mVAN and 10 mVAD, *p* = NS (not significant) by Student's *t*-test. **(C)** The serum retinal levels of offspring at 6 weeks old. Serum VA levels were detected by HPLC. Values represent mean ± SD. *n* = 10 group A and 10 group C, ***P < 0.001 by Student's *t*-test. **(D,E)** The PCA and PLS-DA analysis of the samples between the group A and group C. **(D)** PCA scores were plotted based on the relative abundance of OTUs (*n* = 10). **(E)** PLS-DA was plotted based on the unweighted UniFrac distance metrics (*n* = 10). The red circles represent for the group C, blue squares for the group A. **(F)** The phylum level of dominant bacteria with relative abundance more than 1% in the two groups. **(G)** The genus level of dominant bacteria with relative abundance more than 5% in the two groups. **(H,I)** Different structures of colonic mucosal microbiota in rats with VAN or VAD diet by LEfSe analysis. **(H)** A cladogram of the statistical and biological differences in the colonic microbiota between the group A and group C, which are shown by the color of the most abundant phylotypes (red indicating group C, green group A, and yellow non-significant) (*n* = 10). **(I)** A histogram of the LDA scores for the most abundant phylotypes (*n* = 10).

The offspring in group C were delivered and nursed by maternal VAN rats and then fed a VAD diet for 3 wk after weaning (Figure 3A). The mothers' average serum retinol levels before copulation were 1.48 ± 0.22 μmol/L (group A) and 1.170 ± 0.132 μmol/L (group C), which were not significantly different (*P* > *0.05, t*-test) (Figure 3B). The pups' average serum retinol level in group C was 0.58 ± 0.04 μmol/L, which was significantly decreased compared with that of rats in group A (1.26 ± 0.07 μmol/L) (*P* < *0.001, t*-test) (Figure 3C).

Meanwhile, no significant differences were found between community richness and community diversity in group A and group C, as shown in rank abundance curves (data not shown). Differences in the ACE, Chao1, Shannon, and Simpson indices of the two groups were also not significant (data not shown). PCA analysis results showed that PC1 was 40.49% and PC2 was 15.78% and that the samples of the two groups were concentrated in the center of the coordinate system (Figure 3D). In addition, the R value of ANOSIM was 0.1233 and the *P*-value was 0.038 (Figure 3D). PLS-DA clearly distinguished the two groups based on the direction of the PLS-DA 1 axis, explaining −70.71% of the total variance. Group A had better aggregation than group C, indicating that the group A samples had higher similarity than those of group C (Figure 3E).

As demonstrated in Figure 3F, in group C, predominant phyla with a relative abundance of more than 1% were concentrated in the phyla *Proteobacteria* (48.32%), *Bacteroidetes* (21.57%), *Firmicutes* (19.40%), *Deinococcus-Thermus* (3.21%), *Actinobacteria* (3.00%), *Cyanobacteria* (1.84%), and *Verrucomicrobia* (1.37%). Compared with those in group A, the relative abundances of *Bacteroidetes, Firmicutes, Deinococcus-Thermus, Cyanobacteria*, and *Verrucomicrobia* were increased and the relative abundances of *Proteobacteria, Actinobacteria*, and *Acidobacteria* were decreased in group C, but the differences were not statistically significant (*P*>0.05, Metastats). The *Firmicutes*/*Bacteroidetes* values of group A and group C were 1.21 ± 0.77 and 1.72 ± 0.71, respectively (*P* > 0.05, Student's *t*-test). As shown in Figure 3G, 4 genera accounted for more than 5% of the gut microbiota in group C, namely, *Rhodanobacter* (17.46%), *Hydrotalea* (13.68%), *Burkholderia* (12.54%), and *Bradyrhizobium* (6.8%). Compared with those in group A, the relative abundances of *Rhodanobacter* and *Burkholderia* were increased and the relative abundances of *Hydrotalea, Bradyrhizobium, Allobaculum, Helicobacter* and *Collinsella* were decreased in group C (*P* > 0.05, Metastats).

LEfSe (Figures 3H,I) indicated that significant variations were observed in the composition of the group A and group C samples. There were 9 different genera, namely, *Pelagibacterium* (LDA = 3.1, *P* = 0.02), *Bifidobacterium* (LDA = 2.4, *P* = 0.04), *Simplicispira* (LDA = 2.8, *P* = 0.003), *Aggregatibacter* (LDA = 3.1, *P* = 0.008), *Klebsiella* (LDA = 2.9, *P* = 0.03), *Faecalibacterium* (LDA = 2.9, *P* = 0.02), *Actinotalea* (LDA = 3.2, *P* = 0.04), *Allobaculum* (LDA = 4.4, *P* = 0.03), and *Nesterenkonia* (LDA = 4.1, *P* = 0.04), in group A and 6 different genera in group C, namely, *Christensenella* (LDA = 2.8, *P* = 0.02), *Lysobacter* (LDA = 3.5, *P* = 0.02), *Tetrasphaera* (LDA = 3.08, *P* = 0.02), *Staphylococcus* (LDA = 3.02, *P* = 0.04), *Salinicoccus* (LDA = 2.6, *P* = 0.03), and *Anaerobacillus* (LDA = 3.2, *P* = 0.008). The above analysis found that all the key genera belonged to the predominant phyla.

For further analysis, correlation analysis indicated that the *Faecalibacterium, Pelagibacterium, Simplicispira, Aggregatibacter*, and *Nesterenkonia* were significantly and positively related to serum retinol levels (*P* < 0.05, Spearman test) and that *Tetrasphaera, Lysobacter, Anaerobacillus, Salinicoccus, Staphylococcus, Christensenella* were negatively correlated with serum retinol levels (*P* < 0.05, Spearman test) (Table 1). The remaining significant genera in both groups were not found to have a statistically significant correlation with serum retinol levels.

**Table 1:** The Relationship between the serum retinol levels and the relative rebundance of significant genus in the group A and group C.

**Genus**	**Correlation coefficient(ρ)**	***P-value***	**Relativity**
Actinobacteria-*Tetrasphaera*	−0.517	0.032	Negative correlation
Proteobacteria-*Lysobacter*	−0.496	0.031	
Firmicutes-*Anaerobacillus*	−0.627	0.004	
Firmicutes-*Salinicoccu*s	−0.456	0.505	
Firmicutes-*Staphylococcus*	−0.541	0.017	
Firmicutes-*Christensenella*	−0.486	0.035	
Firmicutes-*Faecalibacterium*	0.505	0.027	Positive correlation
Proteobacteria-*Pelagibacterium*	0.544	0.016	
Proteobacteria-*Simplicispira*	0.539	0.017	
Proteobacteria-*Aggregatibacter*	0.559	0.013	
Actinobacteria-*Nesterenkonia*	0.469	0.034	

### Effect of VAD in the Early-Life Period on the Colonic Mucosal Microbiota of Rats

**Figure 4:**
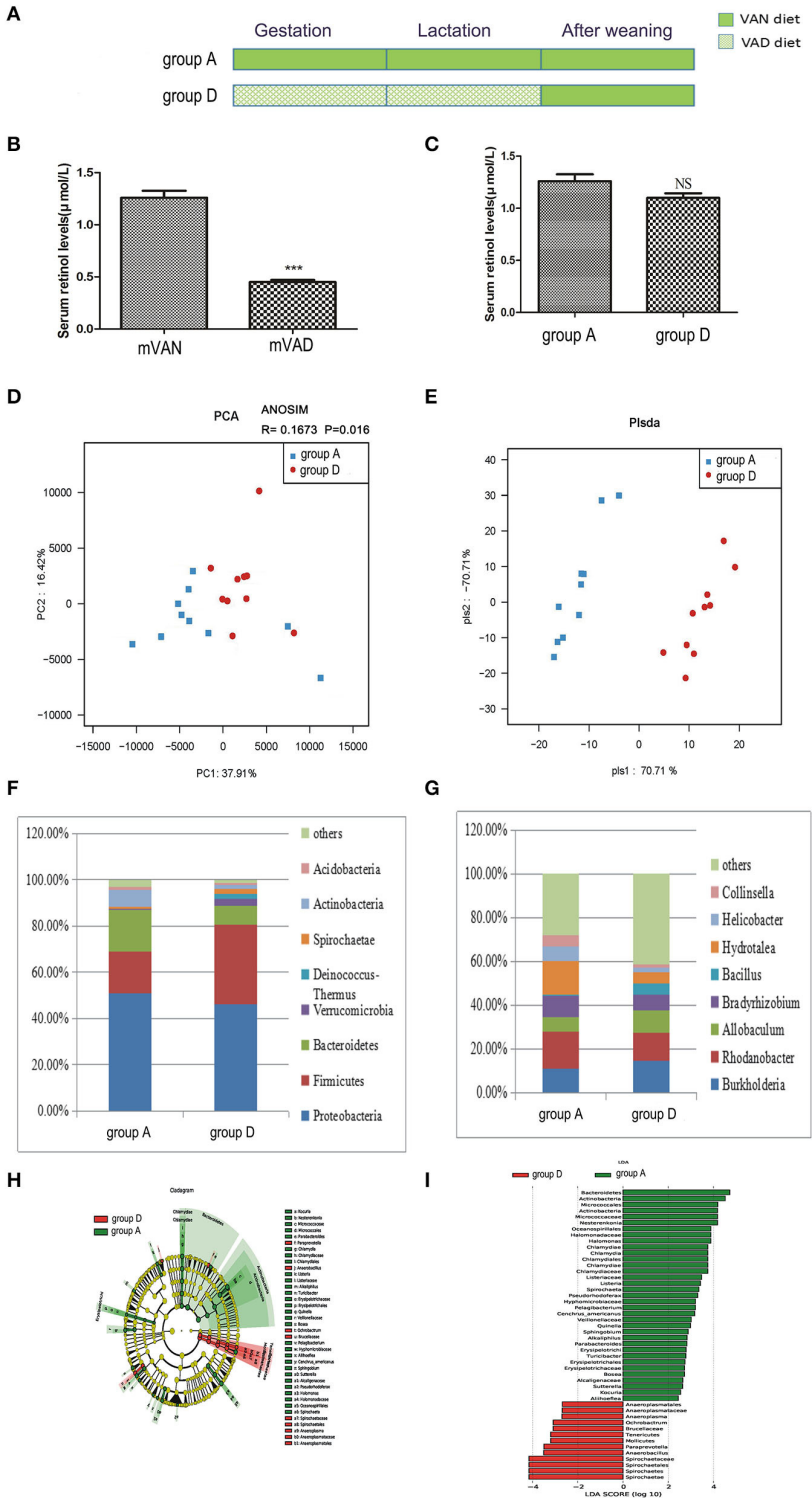
The comparison of colonic mucosal microbiota between the group A and group D. **(A)** Schematic diagram showing the two offspring groups: group A, group D. The group A pups were delivered and nursed by maternal VAN rats and then fed a VAN diet for 3 wk after weaning. The group D pups were birthed and nursed by maternal VAD rats and fed a VAN diet for 3 wk after weaning. **(B)** The serum retinal levels of maternal rats. Serum VA levels were detected by HPLC. Values represent mean ± SD *n* = 10 mVAN and 10 mVAD, ***P < 0.001 by Student's *t*-test. **(C)** The serum retinal levels of offspring at 6 weeks old. Serum VA levels were detected by HPLC. Values represent mean ± SD *n* = 10 group A and 10 group D, *p* = NS (not significant) by Student's *t*-test. **(D,E)** The PCA and PLS-DA analysis of the samples between the group A and group D. **(D)** PCA scores were plotted based on the relative abundance of OTUs (*n* = 10). **(E)** PLS-DA was plotted based on the unweighted UniFrac distance metrics (*n* = 10). The red circles represent for the group D, blue squares for the group A. **(F)** The phylum level of dominant bacteria with relative abundance more than 1% in the two groups. **(G)** The genus level of dominant bacteria with relative abundance more than 5% in the two groups. **(H,I)** Different structures of colonic mucosal microbiota in rats with VAN or VAD diet by LEfSe analysis. **(H)** A cladogram of the statistical and biological differences in the colonic microbiota between the group A and group D, which are shown by the color of the most abundant phylotypes (red indicating group D green group A, and yellow non-significant) (*n* = 10). **(I)** A histogram of the LDA scores for the most abundant phylotypes (*n* = 10).

The pups in group D were delivered and nursed by maternal VAD rats and then fed a VAN diet for 3 wk after weaning (Figure 4A). The mothers' average serum retinol levels before copulation were 1.26 ± 0.07 μmol/L for VAN rats and 0.45 ± 0.02 μmol/L for VAD rats (*P* < 0.001, *t-*test) (Figure 4B). Three weeks after the rats were weaned, the serum retinol levels in both group A and group D were normal, with no significant difference (*P* > 0.05, *t*-test) (Figure 4C).

Rarefaction curves, species richness, species diversity and evenness were analyzed in the communities of the colonic mucosal microbiota in groups A and D, which revealed that there were no significant differences (data not shown). PCA analysis results showed that PC1 was 37.91% and PC2 was 16.42% and that the samples of the two groups were concentrated in the center of the coordinate system. Moreover, most of the samples in group A were biased to the left side, while those in group D trended to the right side, and the R value of ANOSIM analysis was 0.1673 and the *P*-value was 0.016 (Figure 4D). PLS-DA clearly distinguished group A and group D, indicating that there were significant differences between the two groups. However, the aggregation degree of samples in each group was largely similar (Figure 4E).

Figure 4F shows the predominant phyla with a relative abundance > 1%. Compared with those in group A, the abundances of *Proteobacteria* (46.3%) and *Actinobacteria* (1.9%) in group D decreased weeny (*P* > 0.05, Metastats), and the abundance of *Bacteroidetes* (8.1%) in group D was significantly decreased (*P* = 0.03, Metastats), while those of *Firmicutes* (34.3%), *Verrucomicrobia* (3.1%), *Deinococcus-Thermus* (2.3%), and *Spirochaetes* (1.9%) were increased (*P* > 0.05, Metastats). In group A, the abundance of only *Acidobacteria* (1.4%) was higher than that of group D (*P* > 0.05, Metastats).The *Firmicutes*/*Bacteroidetes* values of group A and group D were 1.21 ± 0.77 and 8.34 ± 3.04, respectively (*P* < 0.05, Student's *t*-test). In the gut microbiota genera that were more than 5% at the genus level (Figure 4G), the abundances of *Burkholderia* (14.7%), *Allobaculum* (9.9%), and *Bacillus* (5.3%) in group D were higher than those in group A (*P* > 0.05, Metastats). The abundances of *Rhodanobacter* (12.9%), *Bradyrhizobium* (7.3%), and *Hydrotalea* (5.2%) in group D were lower than those in group A, but only the abundance of *Hydrotalea* (5.2%) had a significant difference (*P* = 0.02, Metastats analysis). However, the relative abundance of *Helicobacter* (6.6%) and *Collinsella* (5.5%) were higher in group A than in group D (*P* > 0.05, Metastats).

LEfSe (Figures 4H,I) indicated that significant variations were observed in the composition of the group A and group D samples. By comparing group A and group D, 17 different genera were found in group A, namely, *Halomonas* (LDA = 3.9, *P* = 0.01), *Chlamydia* (LDA = 3.7, *P* = 0.02), *Quinella* (LDA = 3.0, *P* = 0.001), *Cenchrus_americanus* (LDA = 3.2, *P* = 0.04), *Pelagibacterium* (LDA = 3.2, *P* = 0.003), *Parabacteroides* (LDA = 2.8, *P* = 0.01), *Listeria* (LDA = 3.4, *P* = 0.04), *Bosea* (LDA = 2.7, *P* = 0.03), *Alkaliphilus* (LDA = 2.8, *P* = 0.04), *Aliihoeflea* (LDA = 2.4, *P* = 0.02), *Kocuria* (LDA = 2.5, *P* = 0.04), *Nesterenkonia* (LDA = 4.2, *P* = 0.01), *Spirochaeta* (LDA = 3.4, *P* = 0.04), *Turicibacter* (LDA = 2.8, *P* = 0.04), *Pseudorhodoferax* (LDA = 3.3, *P* = 0.04), *Sutterella* (LDA = 2.6, *P* = 0.01), and *Sphingobium* (LDA = 2.9, *P* = 0.04), and 4 different genera in group D, namely, *Ochrobactrum* (LDA = 3.1, *P* = 0.02), *Anaerobacillus* (LDA = 3.5, *P* = 0.008), *Paraprevotella* (LDA = 3.5, *P* = 0.02), and *Anaeroplasma* (LDA = 2.7, *P* = 0.03).

### Effect of VAD Between Embryonic and Postnatal Stages on the Colonic Mucosal Microbiota of Rats

**Figure 5:**
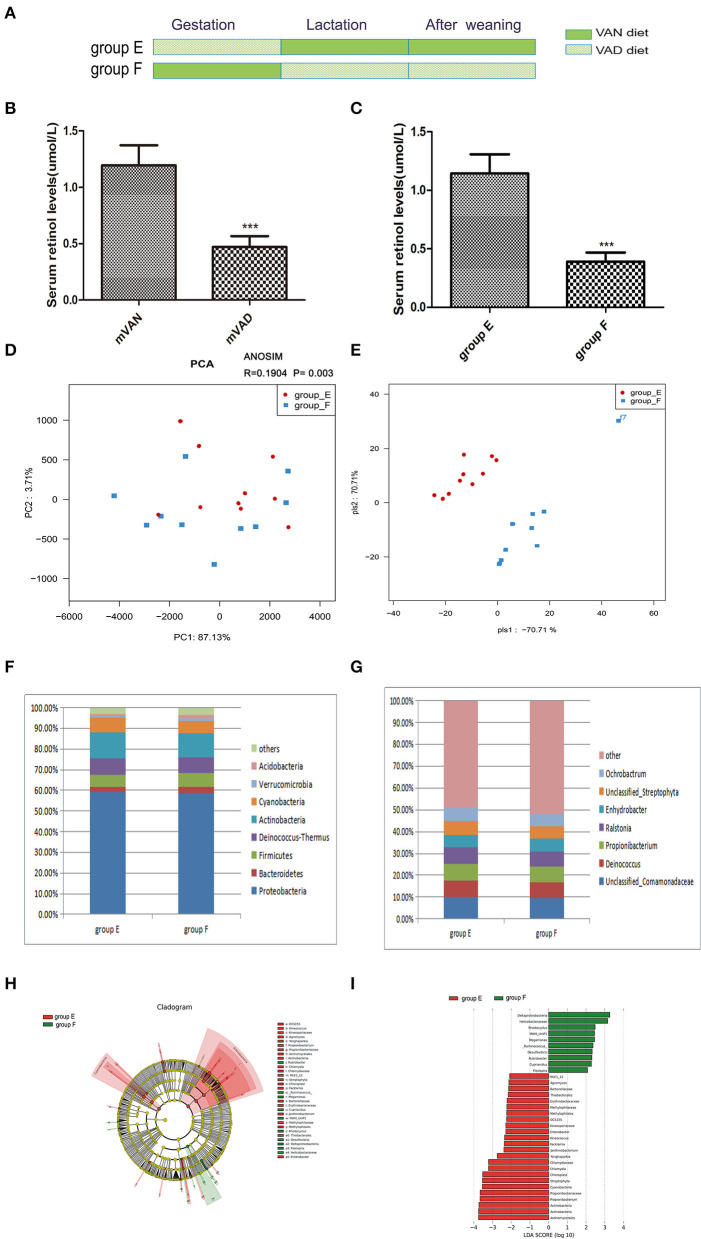
The comparison of colonic mucosal microbiota between the group E and group F. **(A)** Schematic diagram showing the two offspring groups: group E, group F. The group E pups were cross-fostered to VAN dams during the lactation period and then fed the VAN diet after weaning. The group F pups were cross-fostered to VAD dams during the lactation period and then fed the VAD diet after weaning. **(B)** The serum retinal levels of maternal rats. Serum VA levels were detected by HPLC. Values represent mean ± SD *n* = 10 mVAN and 10 mVAD, ***P < *0.001* by Student's *t*-test. **(C)** The serum retinal levels of offspring at 6 weeks old. Serum VA levels were detected by HPLC. Values represent mean ± SD *n* = 10 group E and 10 group F, ***P < 0.001 by Student's *t*-test. **(D,E)** The PCA and PLS-DA analysis of the samples between the group E and group F. **(D)** PCA scores were plotted based on the relative abundance of OTUs (*n* = 10). **(E)** PLS-DA was plotted based on the unweighted UniFrac distance metrics (*n* = 10). The red circles represent for the group E, blue squares for the group F. **(F)** The phylum level of dominant bacteria with relative abundance more than 1% in the two groups. **(G)** The genus level of dominant bacteria with relative abundance more than 5% in the two groups. **(H,I)** Different structures of colonic mucosal microbiota in rats with VAN or VAD diet by LEfSe analysis. **(H)** A cladogram of the statistical and biological differences in the colonic microbiota between the group E and group F, which are shown by the color of the most abundant phylotypes (red indicating group E, green group F, and yellow non-significant) (*n* = 10). **(I)** A histogram of the LDA scores for the most abundant phylotypes (*n* = 10).

To determine whether pregnancy or lactation were the most significant contributors to the differences between the group A and group D microbial communities, we designed group E and group F. The pups of group E were delivered by maternal VAD rats, cross-fostered by VAN dams during the lactation period and then fed the VAN diet after weaning, while the group F pups were delivered by maternal VAN rats, cross-fostered by VAD dams during the lactation period and then fed the VAD diet after weaning (Figure 5A). The mothers' average serum retinol levels before copulation were 1.19 ± 0.06 mol/L for VAN rats and 0.47 ± 0.03 mol/L for VAD rats (*P* < 0.001, *t*-test) (Figure 5B). Three weeks after the pups were weaned, the average serum retinol level of group E was 1.14 ± 0.05 μmol/L, which was significantly increased compared with that of group F (0.39 ± 0.03 μmol/L) (*P* < 0.001, *t*-test) (Figure 5C). Rarefaction curves, species richness, species diversity and evenness were analyzed in the communities of the colonic mucosal microbiota in group E and group F, and these analyses revealed that there were no significant differences (data not shown). PCA analysis results showed that PC1 was 87.13% and PC2 was 3.71% and that the samples of group E were biased to the right side, while those in group F were scattered over the coordinate system (Figure 5D). Meanwhile, ANOSIM analysis showed the R value was 0.1904 and the *P*-value was 0.003 (Figure 5D). PLS-DA clearly distinguished group E and group F, indicating that there were significant differences between the two groups (Figure 5E).

At the taxonomic level of phyla with relative abundance > 1%, the results are shown in Figure 5F. Group E showed a reduced relative abundance of *Bacteroidetes* (2.67%), *Verrucomicrobia* (1.05%), and *Acidobacteria* (0.85%) and an increased relative abundance of *Proteobacteria* (59.11%), *Deinococcus-Thermus* (8.03%), and *Cyanobacteria* (6.84%) (*P* > 0.05, Metastats). However, group E exhibited a significant increase in *Actinobacteria* (12.79%) and a significant decrease in *Firmicutes* (5.73%) (*P* = 0.04, Metastats). The *Firmicutes*/*Bacteroidetes* values of group E and group F were 2.67 ± 0.37 and 3.4 ± 0.49, respectively (*P* > 0.05, Student's *t*-test). In the gut microbiota genera that accounted for more than 5% at the genus level (Figure 5G), we observed that the abundances of *Unclassified_Comamonadaceae* (14.7%), *Propionibacterium* (7.86%), *Deinococcus* (7.71%), *Ralstonia* (7.3%), *Unclassified_Streptophyta* (6.34%), and *Ochrobactrum* (6.02%) in group E were higher than those in group F; however, the abundance of *Propionibacterium* was significantly increased in group E (*P* = 0.04, Metastats). Only the abundance of *Enhydrobacter* (5.97%) was lower in group E than in group F (*P* > 0.05, Metastats). LEfSe (Figures 5H,I) indicated that significant variations were observed in the composition of the E and F groups. There were 7 different genera in group E, namely, *Kineococcus* (LDA = 2.4, *P* = 0.006), *Chlamydia* (LDA = 3.2, *P* = 0.03), *Janthinobacterium* (LDA = 2.4, *P* = 0.01), *Agromyces* (LDA = 2.1, *P* = 0.03), *Yonghaparkia* (LDA = 2.7, *P* = 0.03), *Enterobacter* (LDA = 2.29, *P* = 0.03), and *Propionibacterium* (LDA = 3.7, *P* = 0.03), and 6 different genera in group F, namely, *Flexispira* (LDA = 2.1, *P* = 0.02), *Rhodocyclus* (LDA = 2.5, *P* = 0.03), *Desulfovibrio* (LDA = 2.3, *P* = 0.03), *Ruminococcus* (LDA = 2.4, *P* = 0.04), *Megamonas* (LDA = 2.5, *P* = 0.03), and *Cupriavidus* (LDA = 2.3, *P* = 0.03).

## Discussion

Vitamin A (VA) and its metabolites (retinoic acid, etc.) play a key role in development and are important in the homeostasis of many organs. VA deficiency (VAD) in childhood can lead to a decline in resistance to infection, affecting children's growth and development and increasing the risk of childhood death (21). Retinoic acid (RA) deficiency alters the gut microbiome (22), and RA reduces the ratio of *Firmicutes* to *Bacteroidetes* (23). Previously, our group found that the intestinal mechanical barrier was destroyed and intestinal permeability and intestinal inflammation increased in response to VAD in rats (4).

In the current study, we further observed a down regulation of serum retinal level at postnatal day 7. We speculate that it may be due to the sharp decrease of maternal VA level during gestation, resulting in insufficient storage of both mother and pup. On the other hands, the rapid growth of postnatal pups requires VA, which may be lead to further reduce serum retinal concentration of pups, even if maternal VA level showed a pick up after delivery for 7 days.

PCA is a method to reduce the dimension of multidimensional data and reflect the difference of multidimensional data on the two-dimensional coordinate graph. The more similar the community composition of the samples, the closer their distances in the PCA diagram are (24–26). Anosim analysis is used to test whether the difference between groups is significantly greater than the difference within groups, so as to judge whether the grouping is meaningful. *R* > 0 indicates significant difference between groups (27). Our research results showed that there were no discrete samples in our data, and the *R* values were all > 0, which proves our grouping is meaningful. Based on the results of our study, PCA was difficult to detect the difference between the groups accurately and objectively. PLS-DA can effectively distinguish the observed values between groups, and can find the influencing variables that lead to the difference between groups, so as to avoid the deficiency of PCA (28). Our results showed that there were significant differences in the community composition of the samples in AB, AC, AD, and EF groups.

In terms of ecological imbalance, the ratio of *Firmicutes* to *Bacteroidetes* is a commonly used evaluation index representing the composition imbalance of the intestinal microbiota (29). In the current study, VAD in the different periods, namely, persistent VAD beginning from gestation (group B), VAD after weaning (group C), VAD in early life (pregnancy and lactation) (group D), VAD during pregnancy (group E), and postnatal VAD (group F), altered colonic mucosal microbiota structures. Our data demonstrated that except for the VAD group after weaning (group C), the abundance of *Bacteroidetes* decreased in the remaining VAD groups; however, the ratios of *Firmicutes* to *Bacteroidetes* in all the VAD groups displayed an increase compared to that of group A, especially in group D. Hui-Xin's study found that retinoic acid treated mice had a reduced ratio of *Firmicutes* to *Bacteroidetes* (2-fold increase in *Bacteroidetes*) (23).

The LEfSe is valid to identify the features that explain differences between groups. LEfSe results showed that the abundance of *Bifidobacterium* in group C was decreased significantly, while that of *Staphylococcus* was notably increased, which was significantly and negatively correlated with serum retinol levels. *Bifidobacterium* is beneficial for human health, and many strains of this genera are widely used as probiotics (30). Yuqing Feng et al.'s study has shown that *Bifidobacterium*, as a dominant genus, played an important role in terms of the diversity and robustness of the gut microbiota (31). *Staphylococcus* are gram-positive cocci, most of which are non-pathogenic bacteria. However, when the abundance of dominant bacteria in the intestine decreases, they multiply and produce toxins, which destroys intestinal mucosal homeostasis and increases permeability (32). Compared to either the group B, C or D, LEfSe indicated that significant variations were observed in the composition of the group A. Thesefore, these results make us to speculate that lack of VA throughout the life cycle (pregnancy, lactation and after weaning) may impacted on the composition of the microbial microbiota in the intestinal mucosa.

Intriguingly, as shown in the LEfSe results, the abundance of *Ochrobactrum* in both group B and D were increased significantly compared with that of group A, while not in group C. The genus *Ochrobactrum* comprises a group of non-fermenting, aerobic, Gram-negative bacilli that are environmentally ubiquitous. They are associated with opportunistic central catheter-associated infections in immunocompromised human hosts. Other reports of infections in humans include endocarditis, peritonitis, meningitis, osteomyelitis, endophthalmitis, septic arthritis, and bacteremia (33). The above data further indicates that VA deficiency of early-life stage (gestation and lactation) can enhance of *Ochrobactrum* abundance in the intestinal mucosa, which may increase the risk of infection.

To further study the effects of VAD during pregnancy and lactation, groups E and F were designed. In the present study, *Propionibacterium* was significantly increased in group E. *Propionibacterium* spp. is an anaerobic Gram-positive rod-shaped bacterium, which is commonly found in the pilosebaceous follicles of the human skin, oral cavity, conjunctiva, respiratory and intestinal tract and external ear canal. *Propionibacterium* spp. can be responsible for severe infections including endocarditis, meningitis and brain abscess, endophthalmitis conjunctivitis and osteomyelitis or spondylodiscitis (34). The LEfSe also results showed that the abundance of *Enterobacter* in group E was increased significantly. The genus *Enterobacter* is a member of the coliform group of bacteria. Several strains of these bacteria are pathogenic and cause opportunistic infections in immunocompromised hosts and in those who are on mechanical ventilation (35).

Correlation analysis indicated that the genus *Faecalibacterium* was significantly and positively related to serum retinol levels. In healthy people, *Faecalibacterium prausnitzii* represents more than 5% of the bacteria in the intestine, making it one of the most common gut bacteria (36). Currently *Faecalibacterium prausnitzii* is the only strain in the genus *Faecalibacterium*. *Faecalibacterium prausnitzii* is the most important butyrate-producing bacteria in the human colon (37) and displays a crucial role in producing energy to the colonocytes as well as anti-inflammatory metabolites that cooperate for the intestinal health (38). *Faecalibacterium prausnitzii* has been considered as a bioindicator of human health, once when its population is altered (decreased), inflammatory processes are favored (39).

## Conclusions

In conclusion, VA deficiency in different life periods (gestation, lactation and weaning) led to an imbalance of the ratio between *Firmicutes* and *Bacteroidetes* in the colonic mucosal microbiota of adolescent rats, suggesting that VAD can alter gut microbiota homeostasis. After the lack of VA in early life (gestation and lactation), the abundance of *Ochrobactrum* was increased, and those of opportunistic pathogens such as *Propionibacterium* and *Enterobacter* were increased in the VAD of pregnancy, indicating that VAD in the early-life period may increase opportunistic pathogens to cause infection. So the early-life stage maybe become a time window of VA intervention to improve intestinal microbiota caused by VA deficiency, but the specific mechanism requires more in-depth research.

## Data Availability Statement

The datasets presented in this study can be found in online repositories. The names of the repository/repositories and accession number(s) can be found at: https://www.ncbi.nlm.nih.gov/, PRJNA 615186.

## Ethics Statement

The animal study was reviewed and approved by the Animal Experimentation Ethical Committee of Chongqing Medical University (Chongqing, China).

## Author Contributions

The authors show their grateful appreciation to the participants' diligent commitment in this study. BC and SL were responsible for designing the study, conducting data analysis, interpreting the results, preparing for the submission, and revising the manuscript. JC and TL offered their valued expertise in the laboratory practice. DF and LX contributed to the discussion and data interpretation. WS and TY advised on the statistical analysis. All authors contributed to the article and approved the submitted version.

## Conflict of Interest

The authors declare that the research was conducted in the absence of any commercial or financial relationships that could be construed as a potential conflict of interest.

## Abbreviations

VA: Vitamin A
VAN: Vitamin A normal
VAD: Vitamin A deficiency
HPLC: high-performance liquid chromatography
mVAN: maternal rats with VAN diet
mVAD: maternal rats with VAD diet
pVAN: the pups of the mVAN
pVAD: the pups of the mVAD.
