# Supplementary figures and images for: Vitamin A Deficiency in the Early-Life Periods Alters a Diversity of the Colonic Mucosal Microbiota in Rats
Source: Front Nutr. 2020 Dec 4;7:580780. doi: 10.3389/fnut.2020.580780 (PMC7793871; doi:10.3389/fnut.2020.580780)

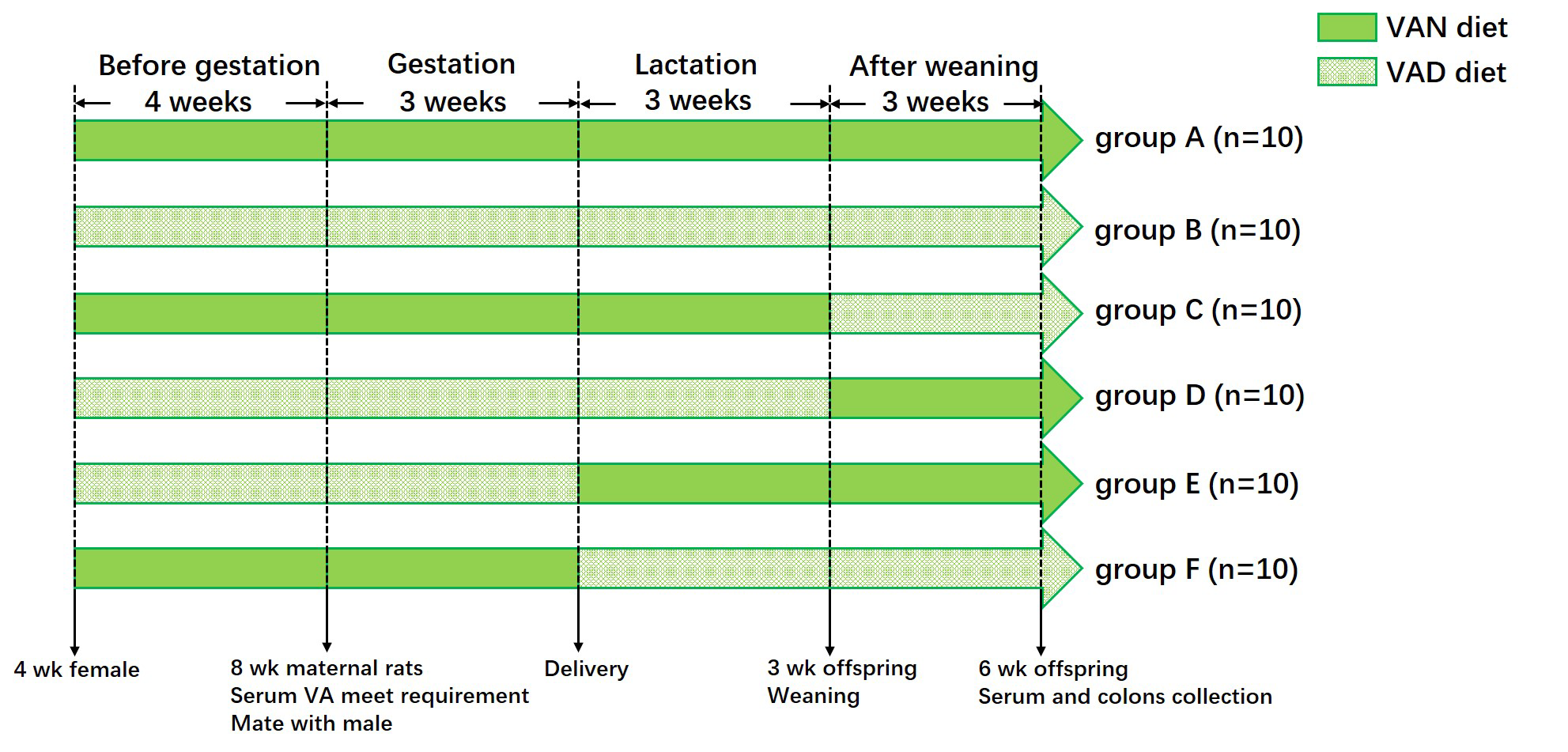

Supplement: Supplementary file 1 [file Image_1.TIF]
